# Molecular Analysis of Repeated Methicillin-Resistant *Staphylococcus aureus* Infections in Children

**DOI:** 10.1371/journal.pone.0014431

**Published:** 2010-12-28

**Authors:** Chih-Jung Chen, Lin-Hui Su, Tzou-Yien Lin, Yhu-Chering Huang

**Affiliations:** 1 Divisions of Pediatric Infectious Diseases, Department of Pediatrics, Chang Gung Children's Hospital and Chang Gung Memorial Hospital, Taoyuan, Taiwan; 2 Pediatric Research Center, Chang Gung Memorial Hospital, Taoyuan, Taiwan; 3 College of Medicine, Chang Gung University, Taoyuan, Taiwan; 4 Department of Laboratory Medicine, Chang Gung Memorial Hospital, Taoyuan, Taiwan; BMSI-A*STAR, Singapore

## Abstract

**Background:**

Methicillin-resistant *Staphylococcus aureus* (MRSA) is a major pathogen that causes severe morbidity and mortality in hospitalized patients. It is unclear whether repeated MRSA infections in pediatric patients are caused by relapse of previous infecting strains or by acquiring new strains from extrinsic sources. The study aimed to define the genetic relatedness of MRSA isolates from children with repeated infections.

**Methodology/Principal Findings:**

Children with multiple MRSA infections during 2004–2006 were identified in a teaching hospital. Repeated infections were confirmed by chart review and the responsible isolates were genotyped and screened for Panton-Valentine leukocidin (PVL) genes. Two consecutive episodes comprised an infection pair, and strain relatedness was defined for each pair as indistinguishable, highly related, or distinct if the isolates were of the same subtype, the same genotype, or different genotype, respectively. A total of 114 episodes comprising 66 infection pairs were identified in 48 children. The interval of infection pairs ranged from 15 days to 346 days, with a median duration of 57.5 days. Genotypings classified all isolates into 7 genotypes and 31 subtypes. Of 66 pairs, 46 (69.7%), 13 (19.7%) and 7 (10.6%) pairs were caused by indistinguishable, highly related and distinct strains, respectively. Subsequent infections caused by indistinguishable strains were more common for PVL-positive strains (17/18, 94.4%) than for PVL-negative strains (29/48, 60.4%, P = 0.007). The strain relatedness was not affected by the durations of interval between infections.

**Conclusions/Significance:**

Most repeated MRSA infections in children are caused by indistinguishable strains even after a long period of interval, suggesting that persistent carriage and relapse of initial infecting strains were responsible for the majority of recurrent MRSA infections.

## Introduction

Methicillin-resistant *Staphylococcus aureus* (MRSA) is a major pathogen that causes severe morbidity and mortality in hospitalized patients [1], [2]. In addition to the increasing methicillin resistance among clinical isolates of *S. aureus*, another growing concern is the emerging community-associated (CA) strains infiltrating hospitals as a major cause of health-care associated (HA) infections [3], [4]. The rapid growing CA-MRSA strains harbor a smaller staphylococcal chromosomal cassette *mec* (SCC*mec*) element, produce more exotoxins (e.g., phenol-soluble modulins, Panton-Valentine leukocidin, etc.), and appear more virulent than traditional HA-MRSA strains [5]–[7]. Evidence further suggests that CA-MRSA as a major nosocomial pathogen may result in increased disease severity and prolonged hospitalization in the infected patients [8], [9]. Accordingly, there is a need of more aggressive measures to control the spread of MRSA in hospitals.

The frequently applied strategies in the control of MRSA infections include hand hygiene of healthcare workers, contact isolation of infected patients, and environmental disinfection [10]. Active surveillance of asymptomatic colonized patients, followed by decolonization and/or cohort care, has also been used to prevent MRSA diseases [11]. However, the efficacy of the screening strategy was inconsistent and may be affected by the condition that most MRSA infections were caused by new strains from extrinsic sources or by original colonized strains [12], [13].

In this study, we analyzed all clinical MRSA isolates from children with repeated infections in a children's hospital, where MRSA was endemic, during a 3-year period. The aims of the study were to elucidate whether the repeated MRSA infections are relapse of the original strain or re-infection by a new strain, and whether the strain relatedness is affected by the interval of infections or other previously unidentified factors. The findings, we hope, may help to develop a more effective strategy for prevention of MRSA diseases in children at risk of repeated MRSA infections.

## Results

During the study period, a total of 82 children with multiple MRSA isolates were identified. Clinical relevance of the isolates was confirmed in 48 children with 2 to 6 discrete episodes of MRSA infection (Table 1). Therefore, a total of 114 episodes (136 isolates) were included for further study. In 16 episodes (13 children), multiple isolates could be found from the respective episode. For the isolates derived from each episode, molecular characterization results indicated that they were indistinguishable from each other. The median duration of intervals between two consecutive episodes was 57.5 days (range, 15 days to 346 days). The duration greater than 6 months was identified in 16 pairs of repeated infection episodes from 15 patients. The longest duration between the recovery of the first and the last isolates from a single patient was 19 months. The common clinical syndromes were wound and lung infections which respectively accounted for 47 (41.2%) and 40 (35.1%) episodes. The detailed characteristics of the repeated infection episodes are displayed in Table 2.

**Table 1:** Information of infection episodes and infection pairs among children with repeated methicillin-resistant *S. aureus* infections.

Infections in each individual	No. (%) of patients	No. (%) of episodes	No. (%) of infection pairs
2 episodes	36 (71)	72 (63)	36 (55)
3 episodes	9 (19)	27 (24)	18 (27)
4 episodes	1 (2.1)	4 (3.5)	3 (4.5)
5 episodes	1 (2.1)	5 (4.4)	4 (6.1)
6 episodes	1 (2.1)	6 (5.3)	5 (7.6)
Total	48 (100)	114 (100)	66 (100)

**Table 2:** Characteristics of repeated methicillin-resistant *Staphylococcus aureus* infections in 48 Taiwanese children.

Characteristic	Total (n = 114)	Infection episode in individuals^1^, No. (%)					
		First (n = 48)	Second (n = 48)	Third (n = 12)	Fourth (n = 3)	Fifth (n = 2)	Sixth (n = 1)
Duration from last episode (days)							
Means ± SD	107.7±100.5	-	112.5±101.2	119.6±111.5	60±72.8	29.5±19.1	31
Median	57.5	-	67	61.5	21	29.5	31
Range	15–346	-	15–346	15–306	15–144	16n43	
Clinical syndromes							
Wound infections	47 (41.2)	19 (39.6)	23 (47.9)	4 (33.3)		1 (50)	
Lung infections	40 (35.1)	15 (31.3)	16 (33.3)	4 (16.7)	3 (100)	1 (50)	1 (100)
Primary bacteremia	10 (8.8)	4 (8.3)	4 (8.3)	2 (16.7)			
CVC-associated infections	7 (6.1)	4 (8.3)	2 (4.2)	1 (8.3)			
Ventriculitis	4 (3.5)	2 (4.2)	2 (4.2)				
Others	6 (5.3)	4 (8.3)	1 (2.1)	1 (8.3)			

Abbreviations: CVC, central vascular catheter; SD, standard deviation;

1 Two and three discrete episodes were identified in 36 and 9 patients, respectively. Four, 5 and 6 episodes were identified in one patient each.

Molecular characterization results indicated that all MRSA isolates belonged to three clonal lineages (ST239, ST59 and ST5) which consisted of 7 genotypes and 31 subtypes (Table 3). PFGE type A were the most predominant type (57.9%), followed by PFGE type D (29.8%) and type C (10.5%). PVL genes were exclusively harbored in isolates of PFGE type D. The distribution of isolation sites differed significantly between major genotypes. Compared to isolates of PFGE types A and F carrying SCC*mec* type III or II (HA genotypes), isolates of PFGE types C, D and AN carrying SCC*mec* type IV or V_T_ (CA genotypes) were more commonly isolated from wound or pus (10.4% vs. 85.1%, p<0.001) but less frequently from sputum (58.2% vs. 2.1%, P<0.001) or blood (13.4% vs. 2.1%, P = 0.045) (Table 3).

**Table 3:** Distribution of isolation sites for 7 clones of MRSA responsible for 114 episodes of repeated infections in 48 children.

Lineages	Patterns	No. of PFGE subtypes	Isolation sites, No. (%) of episodes						
			Total	Wound or pus^1^	Sputum^1^	Blood^1^	CVC	CSF	Others
All		31	114	47 (41.2)	40 (35.1)	10 (8.8)	7 (6.1)	4 (3.5)	6 (5.3)
ST239	PFGE A/SCC*mec* III/PVL−	10	42	6 (14.3)	22 (52.4)	4 (9.5)	5 (11.9)	2 (4.8)	3 (7.1)
ST239	PFGE A/SCC*mec* IIIA/PVL−	4	24	1 (4.2)	16 (66.7)	5 (20.8)	0	0	2 (8.3)
ST5	PFGE F/SCC*mec* II/PVL−	1	1	0	1 (100)	0	0	0	0
ST59	PFGE C/SCC*mec* IV/PVL−	8	12	10 (83.3)	1 (8.3)	0	1 (8.3)	0	0
ST59	PFGE D/SCC*mec* V_T_/PVL+	6	33	29 (87.9)	0	1 (3.0)	1 (3.0)	1 (3.0)	1 (3.0)
ST59	PFGE D/SCC*mec* IV/PVL+	1	1	1 (100)	0	0	0	0	0
ST59	PFGE AN/SCC*mec* IV/PVL−	1	1	0	0	0	0	1 (100)	0

Abbreviations: CVC, central vascular catheter; HA, hospital-associated genotypes; CA, community-associated genotypes; PFGE, pulsed-field gel electrophoresis; PVL, Panton-Valentine leukocidin; SCC*mec*, staphylococcal cassette chromosome *mec*; ST, multi-locus sequence type.

1 Comparing to isolates of PFGE C, D and AN, isolates of PFGE type A and F were isolated more commonly from sputum (p<0.001) and blood (p = 0.045) but less frequently from wound/pus (p<0.001).

The 114 episodes could be further categorized into 66 pairs of repeated MRSA infections (Table 1). Of the 66 infection episode pairs, 46 (69.7%) were caused by indistinguishable strains, 13 (19.7%) were associated with highly related strains, and 7 (10.6%) were caused by distinct strains (Table 4). The detailed PFGE types of MRSA isolates from 12 children with three or more episodes are listed in Table 5. Since these pairs were not independent of each other and the rate of indistinguishable strains can be therefore overestimated, a subsidiary analysis was conducted by including only the first infection pair for each single individual. Among 48 infection pairs in 48 children, 34 (70.8%), 8 (16.7%) and 6 (12.5%) pairs were respectively caused by indistinguishable, related and distinct strains.

**Table 4:** Strain relatedness in 66 pairs of repeated MRSA infection episodes, categorized according to PFGE types of preceding isolates and intervals.

Strain relatedness	No. (%) of patients	No. (%) of episode pairs									
		Total	PFGE types of preceding isolates					Duration of intervals (days)^3^			
			A	C	D	F	AN	15–31	31–90	91–180	>180
Total	48^1^	66	40	6	18	1	1	16	27	7	16
Indistinguishable	34 (71)	46 (70)	25 (63)	4 (67)	17 (94)^2^	0 (0)	0 (0)	9 (56)	18 (67)	6 (86)	13 (81)
Highly related	9 (19)	13 (20)	13 (33)	0 (0)	0 (0)	0 (0)	0 (0)	7 (44)	4 (15)	0 (0)	2 (13)
Distinct	6 (13)	7 (11)	2 (5)	2 (33)	1 (6)	1 (100)	1 (100)	0 (0)	5 (19)	1 (14)	1 (6)

PFGE, pulsed-field gel electrophoresis.

1 One child had 3 infection pairs caused respectively by indistinguishable strains (2 pairs) and highly related strains (1 pair).

2 The rate of paired infections caused by indistinguishable strains was higher for PFGE type D (carrying Panton-Valentine leukocidin genes) than for other PFGE types (P = 0.007, Fisher's exact test).

3 Strain relatedness was not associated with the length of intervals (indistinguishable strains vs. highly related or distinct strains, p = 0.404).

**Table 5:** Detailed pulsed-field gel electrophoresis patterns of MRSA isolates from 12 children with three or more episodes.

Case No.	Episode					
	First	Second	Third	Fourth	Fifth	Sixth
1	A4	A4	A4			
2	A9	A9	A9			
3	D11	D11	D11			
4	D4	D4	D4			
5	A4	A	A			
6	A10	A10	A38			
7	AN	C38	C38			
8	A4	A12	A			
9	A38	A3	A38			
10	A	A	A23	A23		
11	A38	A38	A38	A38	A38	
12	A3	A10	A10	A10	A10	A10

The intervals between each paired infection episodes did not differ significantly for infections caused by indistinguishable strains (118.6±98.2 days) compared to those caused by highly related (69.7±96.7 days, p = 0.0985) or distinct strains (121±126.6 days, p = 0.9593). The incidence of indistinguishable strains was 74% among 50 infection pairs with interval greater than 30 pairs, which did not differ significantly from that of the 16 infection pairs with interval of 30 days or less (56%, p = 0.217). Longer intervals of >6 months were found in 16 pairs of infection episodes, and among them, 13 (82%) were caused by indistinguishable strains. Even for the isolates derived from the infection episode pair of longest durations (>11 months), their PFGE types were also indistinguishable. The lack of an association between the length of infection intervals and strain relatedness was further supported by the analysis of episode pairs distributed among 4 arbitrarily categorized durations (<31, 31–90, 91–180 and >180 days, p = 0.404, Table 4).

The strain relatedness was associated with the genotypes of the preceding isolates. Among the paired infection episodes caused by isolates of the three major genotypes, indistinguishable strains were more common for preceding isolates harboring PVL genes (PFGE type D, 94%) than isolates absent for PVL genes (PFGE type A, 63% p = 0.012; type C, 67%, p = 0.143, Table 4). Among the highly related strains identified in 13 pairs of repeated infection episodes, all were belonged to subtypes of PFGE type A.

## Discussion

Results from this study disclosed that nearly 70% of repeated MRSA infections in children were caused by strains that were genetically indistinguishable from which caused the previous infections. The finding strongly suggested that repeated MRSA infections were frequently caused by relapse of previous infecting strains. The observation was in consistent with two studies addressing the same issue in adult patients [14], [15]. Huang et al reported that the same MRSA strain involved in 76% and 72% of repeated infections and infections following colonization, respectively [14]. The close association between colonization and the subsequent clinical infection has also been demonstrated in infants staying in MRSA-endemic neonatal intensive care units [16]. In the present study, since it was a retrospective analysis in nature and we did not survey MRSA colonization at that time, we could not provide the direct evidence of MRSA colonization in these patients and their association with subsequent clinical infection. However, it is reasonable to speculate that, even after effective treatment for clinical infections, colonization of MRSA might still exist in these patients, although direct evidence was not provided in the present study. The acquisition of new strains from other patients, medical care personnel, closed contacts, family members, pets or the environment, though not uncommon, appeared to play a minor role among the repeated MRSA infections observed in this setting. The results also suggest that infection control by standard precaution to break the patient-to-patient or contaminated device-to-patient transmission may at most prevent only 30% of the repeated MRSA infections. Active identification of MRSA colonization, followed by effective decolonization measures, may be required during the health care of children at risk of recurrent MRSA infection.

The genetic diversity of clinical MRSA isolates is usually limited in a defined region within a short period of time [17], [18]. Our findings could be a mere coincidence due to the spread of a few endemic clones prevailing in this institute or the neighboring communities where the pediatric patients came from. Indeed, as noted in the genotyping analysis, there were 3 predominant PFGE clones that belonged to 2 genetic lineages and accounted for the majority (98.2%, Table 3) of repeated MRSA infections in this children's hospital. However, our previous molecular epidemiology studies disclosed that, by PFGE analysis, MRSA in this hospital could be categorized into at least 78 subtypes during the same period of time [19]. In this study, we also identify a total of 31 subtypes among the MRSA isolates studied. The discrimination of the related strains was further increased by the additions of SCC*mec* typing (Table 3). The likelihood of repeatedly getting an identical strain from the exogenous environment should be much less than from a strain which has been persistently carried by the patients. We believe by applying the stringent criteria (same PFGE and SCC*mec* types) of defining the same strain should greatly avoid the bias caused by clonal spread.

It is intriguing that the rate of indistinguishable strains in repeated MRSA infections caused by PVL-positive isolates is significantly higher than that caused by PVL-negative isolates. PFGE type A (ST239) with SCC*mec* III or variants is among one of the six pandemic clones, known as Hungarian clone [20], and has been the most prevalent HA-MRSA clone circulating in major hospitals in Taiwan since early 1990s [21]. PFGE types C and D (ST59 lineage) are both emerging CA-MRSA clones in Taiwan and the PFGE type D/SCC*mec* V_T_/PVL-positive clone has become the most predominant cause of *S. aureus* infection in previously healthy Taiwanese children [22], [23]. It is unclear why with repeated infections the strain relatedness was correlated with genotypes of preceding isolates. It has been suggested that PVL-positive *S. aureus* strains expressed more abundant cell wall-anchored proteins (e.g., staphylococcal protein A, serine-aspartate repeat proteins C and D) and exhibited a greater affinity to extracellular matrix than did PVL-negative strains [7], [24]. Whether PVL-positive MRSA strains possess a greater potential to persistent colonization and facilitate the subsequent relapse infection needs further studies.

The cut-off period of defining distinct infection episodes may have impact in strain relatedness in repeated infections. For instance, a subsequent infection occurred closed to the cessation of antibiotic treatment of prior infection may suggest relapse due to inadequate treatment and increase the incidence of finding the same strains in two episodes. However, the indistinguishable strains in the current study accounted for 56% of subsequent infections occurring between 14 and 31 days. The rate was not greater than those with longer durations of interval (67%, 86% and 81% for interval of 31–90, 91–180 and >180 days, respectively). We also noted that the strain relatedness was not affected by the durations of infection intervals. The data indicated that different cut-off period of defining a new episode did not substantially affect the results or change our conclusion. The finding was compatible with another study addressing this issue in adult populations [14]. For the 46 pairs of repeated infections caused by indistinguishable strains in the present study, 28.3% had intervals greater than 180 days with a maximal duration of 340 days. The long-term carriage of MRSA was also evident in a recent study investigating the duration of MRSA colonization in an adult population [25]. Nearly 50% of the MRSA-carried patients remained colonized in the first year. Since every episode of MRSA infection in our study had been managed with a complete course of antibiotic treatment, the observation might indicate that systemic antibiotic treatment was not sufficient in eradicating MRSA colonization and the immunity against MRSA was incapable of protecting the individual from repeated infection by the same MRSA strains.

There were limitations in this study. Although the indistinguishable strains in infection-infection pair strongly suggested persistent carriage of the same strain to be responsible for MRSA repeated infections, direct evidence by exploring the strain relatedness in colonization-infection pairs was not available in this study with a retrospective-based design. A prospective study incorporating multiple centers and larger patient cohorts will be needed to directly address this issue.

In conclusion, we demonstrated that the majority of repeated MRSA infections in children were caused by the same strains that caused the previous infections even after a long period of intervals. The observation provides new insights into the prevention of MRSA infection. Interruption of transmission may not be sufficient in preventing most of the repeated MRSA infections. Strategies to identify MRSA colonization followed by successful decolonization may be required in children with high risks of MRSA infections.

## Materials and Methods

### Ethics statement

The study was approved by the institute review boards from Chang Gung Memorial Hospital, which allowed retrieve of the patients list from the electronic microbiology database, review of the medical information and characterizations of the responsible isolates. A waiver of consent was granted given the retrospective nature of the project and anonymous analysis of the data.

### Case enrollments

Between January 1, 2004 and December 31, 2006, all clinical isolates of MRSA were collected from the clinical microbiology laboratory of Chang Gung Children's Hospital and stored at −80°C in the research laboratory until use. Information regarding the name and chart number of each patient and the specimen date and source of the respective MRSA isolate was recorded in the hospital's central computer system. In this study, a list of children that were younger than 18 years old and had at least two positive cultures of MRSA was retrieved from the database. If the isolates were not recovered from sterile sites, clinical validation would be performed by reviewing the medical records, aiming to differentiate between MRSA colonization and clinical infection. To define the lung infections, the age-specific clinical criteria for diagnosis of pneumonia was adopted from CDC/NHSN [26], which included a positive chest radiology (e.g. new or progressive and persistent infiltrate, consolidation, cavitation or pneumatoceles in infant) and definitive clinical signs or symptoms (e.g. fever, leucopenia or leukocytosis, new onset or change character of sputum, new onset or worsening cough, dyspnea, rales or bronchial sounds, worsening gas exchange). The diagnosis of wound infections also followed the CDC/NHSN criteria for either surgical sites infection or skin and soft tissue infection where appropriate [26].

### Definitions of infection “episodes” and infection “pairs”

For those being determined as true infections, if the associated isolates were derived consecutively with an interval greater than 14 days and a full course of appropriate antimicrobial agents as judged by the in-vitro susceptibility testing were administered, the infections would be considered as distinct infection episodes. Multiple isolates can be identified from different specimens in single episode. Patients with at least 2 separate infection episodes were enrolled for further analysis. Two consecutive episodes comprised a pair of repeated infection. Children with 2 episodes were considered to have one pair of repeated infection. Those with 3 episodes were considered to have 2 pairs of repeated infection (one pair for the first and the second episodes and the other pair for the second and the third episodes) and so on.

### Molecular characterizations

All MRSA isolates recovered from the enrolled patients were subjected to molecular characterization. Pulsed-field gel electrophoresis (PFGE) of *SmaI-*digested macro fragments of the respective chromosomal DNA was used to fingerprint the isolates according to methods described previously [19]. The DNA fingerprints generated by PFGE were manually analyzed according to the criteria proposed by Tenover et al [27]. Isolates with PFGE patterns differed by 4 or more than 4 bands were considered distinct and designated as different genotypes. Isolates with identical PFGE patterns or differed by less than 4 bands were considered indistinguishable or highly related and defined as the same genotype or a subtype of an existing genotype, respectively. Genotypes were designated consecutively in an alphabetical order and subtypes of a genotype were labeled with Arabic number suffixes. For consistency, genotypes described in the present study followed those described in our previous reports [19], [28]. Only those newly identified PFGE patterns would be assigned with a new genotype name.

The SCC*mec* types I–IV were determined by a multiplex PCR system, whereas type V or V_T_ was determined by the detection of *ccrC* complex as described previously [29], [30]. Control strains for the SCC*mec* typing were kindly provided by Dr. K. Hiramatsu and listed as follows: type I, NCTC10442; type II, N315; type III, 85/2082; type IVa, JCSC4744 and type V, WIS. The control strain for SCC*mec* type V_T_ was provided by Dr. Chih-Chien Wang at Tri-Service General Hospital, Taipei, Taiwan.

Multilocus sequence typing (MLST) was performed among representative strains of major PFGE types according to the instruction provided in the MLST website (http://www.mlst.net). Briefly, each allele was assigned a number by comparing the respective sequence with those of the known alleles in the *S. aureus* MLST database. The allele numbers at each of the seven loci defined the allelic profile of each isolate. An allelic profile was defined as a sequence type (ST).

Panton-Valentine leukocidin (PVL) has been considered an epidemiological marker for CA-MRSA strains. All isolates were investigated for the presence of the *lukS-PV* and *lukF-PV* genes encoding PVL using a single PCR method as previously described [22].

### Determination of genetic relatedness among isolates derived from consecutive infections

Strain relatedness was investigated in isolates accounting for each pair of infection episodes. Isolates derived from a pair of repeated infection were interpreted as indistinguishable if they had the same PFGE patterns as well as the same SCC*mec* elements. Highly related strains were defined if their PFGE patterns were subtypes of the same genotype and they carried the same SCC*mec* elements. Distinct strains were defined if they had different PFGE patterns or carried different SCC*mec* elements.

### Statistical analysis

The descriptive statistics were analyzed with SAS 9.1 software (SAS Institute Inc. Cary, NC) for Windows. Categorical variables were compared by chi-square or Fisher's exact test, when appropriate. Differences in means were assessed by the Student *t*-test. Statistical significance was deemed to be p<0.05.
